# “I Am Interested!”: The Voices of the Community and Their Participation in Health Advisory Boards

**DOI:** 10.1089/heq.2022.0206

**Published:** 2024-01-08

**Authors:** Sylk Sotto-Santiago, Sarah Wiehe, Gina Claxton, Gavin Stamper, Lindsey Delp, Brenda Hudson, Dustin Lynch, Sharon Moe

**Affiliations:** Indiana Clinical and Translational Sciences Institute, Indiana University School of Medicine, Indianapolis, Indiana, USA.

**Keywords:** community advisory board, community engagement, health equity, volunteer registry

## Abstract

**Introduction:**

Researchers can often be challenged by meaningful efforts to involve the public and communities in research. Community and health advisory boards (HABs) offer an opportunity to create a fully intentional and honest relationship between researchers and the community.

**Objective:**

Most recently, the All Indiana (IN) for Health HAB had four openings and a call was published to our community of over 13,800 individuals in the All IN for Health newsletter. Four hundred eighty-eight individuals submitted applications to become part of the board. In what follows, we share the lessons in motivations and interests of individuals who responded.

**Methods:**

The application process included the following questions: What lived experiences and/or personal interests have motivated you to be involved in All IN for Health? Please explain why you are interested in being an All IN for HAB member. Our analysis approach was qualitative and centered on narrative research.

**Results:**

We organize the findings in two categories: Motivation and Interests. Individuals were motivated to participate based on family or friend diagnosis, personal diagnosis, roles as caregivers, desire to impact change and advocacy, role as health professional, and previous participation in research. Interests followed similar themes beginning with crediting their interest to a diagnosis. In addition, we categorized desire to share their experience, personal positionality, and previous research experience, and contributing to the education of student/trainee.

**Conclusion:**

By understanding motivations, we understand needs. This information can be used for other advisory boards, as well as recruitment into research participation and health care advocacy.

## Introduction

All forms of community engagement call for bidirectional communication and relationships between researchers and the local community.^1^ It is important to understand community health needs and the depth from which researchers understand these needs helps to address and focus our priorities.^1^ Researchers can often be challenged by meaningful efforts to involve the public and communities in research.^2^ Community and health advisory boards (HABs) offer an opportunity to create a fully intentional and honest relationship between researchers and the community. Moreover, they offer invaluable consultation to promote healthy communities and foster meaningful research.^2^ HAB can also formalize academic-community partnerships to ensure appropriate bidirectional values and guarantee public representation, while prioritizing the voice of individuals in the community.^3^

An initiative of the Indiana Clinical and Translational Science Institute, All Indiana (IN) for Health, is an online platform that promotes health and research literacy, seeking to increase the public's understanding of the role and value of research in improving health. All IN for Health shares health resources, disseminates research findings, and offers opportunities to participate in the state-wide research volunteer network. Goals of the program include All IN for Health as a trusted resource for health information in Indiana; people of Indiana actively involved in health research; ultimately leading to a future where the population of the state is healthier and happier.

All IN for Health's Advisory Board is currently formed by 20 individuals in the community, some whose interests are also tied to community health organizations they may work with, but most individuals representing the rich diversity and interests of our community. HAB members represent key counties in the state of Indiana with diverse backgrounds, employment, roles and areas of expertise, and interests. HAB participants speak from personal experience. This has been an active board providing advice on strategic directions, active feedback to all efforts, while contributing ideas, priorities, and most importantly, accountability. For example, the HAB has provided important feedback about All IN for Health strategic plan and potential collaborations with industry-community health partnerships.

During the Covid-19 pandemic, the HAB was a critical part of our vaccination campaign. Members shared stories about their own vaccination experiences and helped develop vaccination education materials. Throughout the years, board members have provided feedback on strategic initiatives such as our social media campaign. They have contributed stories and profiles for the newsletter and spoken about All IN for Health at different public events, such as health fairs and within their own communities. Most recently, members answered the call to represent All IN for Health at meetings where partnerships with industry and community health organizations were being developed.

Finally, investigators in need of recruitment materials feedback can also engage the board members as partners in research. Most recently, the HAB had four openings and a call to our registry and community of over 13,800 individuals was published in the All IN for Health newsletter. Four hundred eighty-eight individuals submitted applications to become part of the board. Criteria for HAB participation include diversity of state counties represented and identified as priority areas for the Indiana Clinical and Translational Sciences Institute (Indiana CTSI). At the time, applications did not request information about age, gender, race, and ethnicity. We plan to collect this information in the future. Candidate review and selection are done by the All IN for Health leadership team with final approval from the HAB.

All IN for Health leadership includes its director, associate program director of operations, director of recruitment concierge services, and digital communication and community engagement specialist. Every member of the All IN for Health is also part of the Indiana CTSI and shares effort and time with the institute. In what follows, we share the lessons in motivations and interests submitted by individuals who responded. We offer these to not only reemphasize the importance of HABs but also to highlight potential constructs that increase participation by community members in research, and the message and intention that must exist by researchers, especially within academic-community partnerships Table 1.

**Table 1.** Demographics of All Indiana for Health

Self-reported race/ethnicity		Self-reported gender		Self-reported age	
Hispanic/Latinx	447	Female	8517	0–17	729
Black/African American	1231	Male	3018	18–35	4571
American Indian/Alaska Native	138	Transgender	31	36–64	6149
Native Hawaiian and Pacific Islander	23	Choose not to answer	377	65+	1394
White	10118				
Choose not to answer	886				

## Methods

Four hundred eighty-eight individuals submitted applications for participation in the All IN for HAB. The call for nominations appeared in the All IN for Health newsletter, which includes volunteers of the research network. The volunteer research network comprised community members across the state. The call mentioned a nominal compensation of $50/h, and time commitment of 2-h board meetings through video conferencing every 3 months. Review of these applications for participation in the HAB was deemed exempt from human subject research regulations by the institutional review board).

The application process included the following questions:

(1) What lived experiences and/or personal interests have motivated you to be involved in All IN for Health?
(2) Please explain why you are interested in being an All IN for HAB member.

The data used for this analysis codified individual names to protect confidentiality and only provided responses to All IN for Health's call. Our analysis approach was qualitative and centered on narrative research. Narrative research can be defined as collecting and analyzing the accounts of individuals describing experiences and offer an interpretation.^4^ Narratives detail unique experiences and perceptions and can provide insight on human interaction, conduct, and perceived roles.^4^ Even more so, narrative research can deliver how individuals construct an understanding of an event.

We used an inductive approach to generating codes and themes and analyzed data using the constant comparative method, in which essential concepts were coded and compared over time to extract recurrent themes. Two authors independently read 488 responses to generate codes, also creating thematic categories. We met to discuss data interpretation as a group.

## Results

Narrative analysis keeps the individual's complete story intact as the individual piece of data. The results and discussion in subsequent sections practice restorying as a way to honor individuals' voices.

We organize the findings in two categories: Motivation and Interests. These two categories map the questions asked of potential candidates to the HAB. The motivation category refers to individual's motivation to be part of the All IN for Health initiative. Interest category referred to individual's specific interest in participating in the All IN for HAB.

Individuals were motivated to participate based on (1) family or friend diagnosis, (2) personal diagnosis, (3) roles as caregivers, (4) desire to impact change and advocacy, (5) role as health professional, and (6) previous participation in research. Interests followed similar themes beginning with crediting their interest to a (1) family or friend diagnosis. In addition, the interest of contributing to a (2) greater good was prevalent across the narratives. The role of caregiver (3) was also very present. In addition, we categorized (4) desire to share their experience, (5) personal positionality, (6) previous research experience, (7) education of student/trainee, and (8) previous experience on other boards. Table 2 summarizes these findings.

**Table 2.** Motivation and Interests

Themes: motivations	Themes: interests
Reasons in order of prevalence	
(1) Family/friend diagnosis (2) Personal diagnosis (3) Caregiver	(1) Family/friend diagnosis (2) Greater good (3) Caregiver
(4) Community involvement impacting change and advocacy (5) Health professional (6) Previous participation in research	(4) Sharing experiences (5) Personal positionality (6) Previous experience in research (7) Student/trainee (8) Previous experience in other boards (9) Compensation^a^

^a^ *n*=1.

We share the narratives honoring individual stories for the top 3 motivations and interest. Supplemental material offers a more extensive sample of narratives. To begin, below are representative sample narratives that credit a friend or family member's impactful diagnosis:

### Diagnosis

“I believe in scientific research and if I can personally aid in that aspect, I'm more than willing to give of my time and body. Also, I had a brother that was diagnosed, and succumbed to frontal temporal lobe dementia and provided respite care.”

“Cancer has claimed far too many lives of my family members, starting with my mother when she was only 32 and I was 3. My husband lives with type 2 diabetes along with far too many medications in my opinion at the age of 55. I see a society that just wants a pill instead of lifestyle changes.”

### Caregivers

The role of caregivers for the physical care and emotional support of someone who can no longer care for themselves was also a top 3 reason across the narratives.

“I was a caregiver in two instances that were fatal. To offer the kind of support I wish I had had with my husband and mom is a goal.”

“Having been a care giver for my mother and stepfather, I know the importance of information and support. Sharing information with other care givers and adding support for others is very important to me.”

### Greater good

What is the “greater good?” We refer to utilitarian ethics in this theme, an action that increases net happiness when everyone is considered. In our case, the benefit of the public as more important than oneself.

“I believe that it's part of our civic duty to help our neighbors and I feel that my experience as a mother, spouse of someone with a chronic illness, and sister of someone with a disability could be very helpful.”

“I am interested in helping restore trust from health providers.”

“I believe we all have the opportunity to support medical advancements. As a healthy individual, I believe it is all the more important to donate my time or participated in studies with the hope that participation can help inform our medical community with evidence and knowledge needed to move medicine forward. While I have only participated in a few studies, I am passionate about health equity, and want to find new ways, outside of my professional career, to aid in achieving this critical need” Table 3.

**Table 3.** Representative Quotes

Theme	Quotes
Greater good	“I am interested in becoming an All IN for Health Advisory Board because I am passionate about improving the health of all Hoosiers and making Indiana one of the healthiest states to live in. I work in healthcare (non-clinical) and I am inspired to learn how we can continue to remove barriers for those seeking healthier lives.” “I would like to be a part of health initiatives for our state.” “I would like to be involved in what is going on in my city.”

### Health equity

We also acknowledge specific narratives about the state of health equity in relation to race and ethnicity. For example:

“I am interested in participating as a community member and seeing how ethnic persons experience may differ than an American experience. My experience with the health care community and how my family was treated.”

“I am a Black woman and know that African Americans are underrepresented. I want to be helpful to increase the participation of people of color in studies.

“I want to help change the way minorities are treated in health care.”

**FIG. 1.**
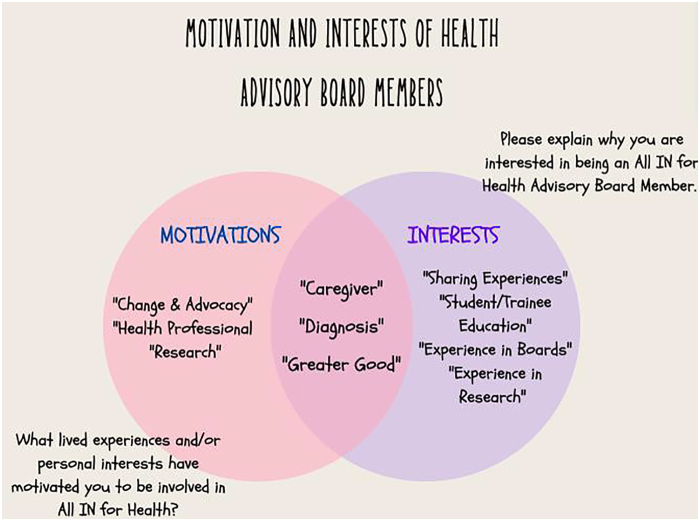
Motivations and interests.

To understand individuals' thoughts, reflections, motivations, and interests, we must look at context. Individual narratives described their relationship with science and medical research (Fig. 1).

## Discussion

Health Advisory Boards connect researchers with communities. Current literature describes best practices in establishing and operating HABs.^3,5,6^ For example, best practices include clarifying purpose, function, and roles; determining HAB membership composition and recruitment strategies; establishing operations, leadership, balancing power, and decision-making; and sustainability.^3^ The contributions of community board members provide critical information from research design, instrument development, and recruitment to result dissemination; however, we must provide a space for this. This co-construction of education, information, and shared leadership equips not only individuals and researchers but also the organization and interests they represent.

In the individual narratives, we learn five main areas of motivation: diagnosis, caregiver roles, change and advocacy, health-affiliated profession, and previous participation in research. Diagnosis close to the individual is the most powerful reason and motivation for participation in HABs. Second, the role of caregivers appears to be also of significance. Caregivers' responsibilities are continuous and can often mean limited time for other activities. It seems like the emotional strain can lead to an even higher connection to offer their perspective for the good of others in the same position or those impacted by a health diagnosis. It may seem that participation in HAB also aligns with patients' motivation for clinical trial participations, which include potential personal benefit and altruism.^7^

Even though compensation was only mentioned by one candidate, it is important to remember the importance of valuing HAB member time. Moreover, we cannot ignore that HABs may result in high turnovers.^5^ The continued engagement of members is the principal factor in the sustainability of HABs and efforts. Therefore, it is imperative that we understand the motivations, lived experiences, and interests of members. Ignoring these would soon result in failure. These motivations represent needs of the community as many individuals speak on their experience with the health care system as a community member. An HAB provides a focus for research efforts, an ongoing partnership to address community health concerns, and a mechanism for building capacity in the community and the academic institution.^3^

Intentional engagement of patients, care givers, and members of the public in health research is recognized as an approach for bridging the gap between research and healthy communities.^8^ Community engagement through HABs commits to research conducted ‘with’ and ‘by’ the individuals it is meant to support. By increasing the relevance and impact of research for those who stand to benefit from its findings, community members contribute experiential knowledge, unique perspectives, and skills that are essential when designing, implementing, and disseminating health research.^9^

## Conclusion

Understanding individual motivations is a critical aspect of the success and effectiveness of HABs. By understanding what drives each individual, whether it is a personal or professional experience, or a commitment to the greater good, organizations can tailor their approach, engagement initiatives, and strategies in authentic ways. The information presented can be used by other advisory boards to enhance board recruitment. It is important to share successes and challenges related to the processes of forming, operating, and maintaining effective community advisory boards. By doing this, we promote ongoing learning and provide a frame of action for continued community-driven actions and research.^3^

### Narrative poem

We would like to conclude with a poem using individuals' narratives. The best way we can think of honoring their commitment and demonstrate the heart in their voice.

My community, my mom, my father,

My brother is hurting.

I am hurting.

Diseases and illness have claimed too many lives.

I want to help.

My identities are vast.

I am a veteran, a caregiver, and a health professional.

I am transgender, low income, a teacher, and a student.

I am a patient, a Black woman, and a parent.

I have worked and I have tried.

Above all, I have strong ties to this community.

My voice is valuable to these discussions.

I want to change the way minorities are treated.

All Hoosiers deserve reliable health care and adequate representation in research.

I want to be helpful.

I want to help.

I love helping.

I am interested.

## Abbreviations Used

CTSI: Clinical and Translational Sciences Institute
HABs: health advisory boards
IN: Indiana
